# In Vitro Elution of Gentamicin from CERAMENT® G Has an Antimicrobial Effect on Bacteria With Various Levels of Gentamicin Resistance Found in Fracture-related Infection

**DOI:** 10.1097/CORR.0000000000002975

**Published:** 2024-01-30

**Authors:** Hans Bezstarosti, Esther M. M. Van Lieshout, Maartje J. B. Van den Hurk, Kirsten Kortram, Pim Oprel, Birgit C. P. Koch, Peter D. Croughs, Michael H. J. Verhofstad

**Affiliations:** 1 Trauma Research Unit, Department of Surgery, Erasmus MC, University Medical Center Rotterdam, Rotterdam, the Netherlands; 2 Department of Pharmacy, Erasmus MC, University Medical Center Rotterdam, Rotterdam, the Netherlands; 3 Department of Medical Microbiology, Erasmus MC, University Medical Center Rotterdam, Rotterdam, the Netherlands

## Abstract

**Background:**

Fracture-related infection is a serious complication after trauma. CERAMENT® G combines dead-space management with local release of gentamicin in a single-stage procedure. Bacterial resistance against antibiotics is increasing. The local effect of CERAMENT® G on bacteria resistant to systemically administered gentamicin is unknown.

**Questions/purposes:**

(1) What is the in vitro elution pattern of gentamicin from CERAMENT® G using a full washout model? (2) What is the in vitro antimicrobial activity (zone of inhibition) of CERAMENT® G against bacterial isolates found in fracture-related infection with different susceptibility levels toward gentamicin?

**Methods:**

Elution of gentamicin from CERAMENT® G was determined in vitro over a period of 2 months. Elution experiments were performed in fivefold, with gentamicin being sampled in threefold at 19 different timepoints within 2 months. Antimicrobial activity was determined using the four most-frequently cultured bacterial species found in fracture-related infection: *Staphylococcus aureus, Staphylococcus epidermidis, Pseudomonas aeruginosa,* and *Enterobacter cloacae*. For each of the species, four different isolates with a different susceptibility to gentamicin were used. According to the European Committee on Antimicrobial Susceptibility Testing, the susceptibility of each isolate was classified into four different groups: fully susceptible (minimum inhibitory concentration 0.064 to 4 mg/L), minimally resistant (minimum inhibitory concentration 4 to 16 mg/L), moderately resistant (minimum inhibitory concentration 8 to 96 mg/L), and highly resistant (minimum inhibitory concentration 24 to 1024 mg/L), depending on each organism. The antimicrobial activity of CERAMENT® G was determined according to the European Committee on Antimicrobial Susceptibility Testing disk protocol. The experiment was performed in fivefold for each isolate. The zone of inhibition was compared between each bacterial isolate and within each of the four separate species. Nonlinear regression statistics were calculated between the zone of interest and logarithmic minimum inhibitory concentration for each bacterial species.

**Results:**

After 24 hours, 95% of all available gentamicin was eluted, and gentamicin was still detectable after 2 months. CERAMENT® G showed antimicrobial activity against all bacterial species; only S*taphylococcus aureus* (with a minimum inhibitory concentration > 1024 mg/L) was not susceptible. The zone of interest of the different bacterial isolates was correlated with the logarithmic minimum inhibitory concentration.

**Conclusion:**

CERAMENT® G offers a bone substitute capable of releasing high levels of gentamicin within a short period of time. This study shows that CERAMENT® G has antimicrobial activity against bacterial isolates that are resistant to gentamicin when systemically administered. This finding raises the question of whether European Committee on Antimicrobial Susceptibility Testing cutoff points for systemic application are useful for the use of local CERAMENT® G. Standardized experiments to determine local antibiotic antimicrobial activity in fracture-related infection treatment are needed to form guidelines for the use of local antibiotics and ultimately improve fracture-related infection treatment.

**Clinical Relevance:**

Local concentrations of gentamicin with CERAMENT® G are much higher than when systemically administered. It seems effective against certain bacterial strains that are not affected by systemically reachable concentrations of gentamicin. CERAMENT® G might still be effective when bacteria that are resistant to systemically administered concentrations of gentamicin are occulated from patients with fracture-related infection.

## Introduction

Fracture-related infection (FRI) is a dreadful complication resulting from orthopaedic and trauma surgery; it increases healthcare costs and compromises patient outcomes [11]. The basic treatment principles of FRI are extensive debridement of infected bone, dead-space management, and antibiotic therapy [18, 20]. Local application of antibiotics can achieve a high local tissue concentration without any systemic side effects and is widely used in the treatment of FRI. However, the use of local antibiotics remains controversial. Recent research shows a potential benefit in preventing FRI in the treatment of open fractures, but good-quality studies researching the effect of local antibiotics in FRI treatment are not available [1, 8, 21]. Because many bacteria begin producing biofilms within hours after inoculation to protect themselves against environmental hazards [9], the concentration of antibiotics must be at its peak during this initial period. The aim of locally applied antibiotics is to create the highest possible local concentration of the antibiotic agent before a biofilm is formed.

A multitude of absorbable antibiotic carriers are available. One commercially available carrier is CERAMENT® (BONESUPPORT), a bone void filler consisting of calcium sulphate and hydroxyapatite [14, 19]. It is readily infused with gentamicin (CERAMENT® G) and can be used to completely fill a bone defect, minimizing any dead space that is filled with blood or serous fluid in a single-stage procedure. The elution kinetics of CERAMENT® G have been studied in vitro, but the model showed a longer half-life of gentamicin compared with clinical models [25].

The largest patient series treated with CERAMENT® G in FRI has been described by McNally et al. [19], who presented a very high success rate with no relapse of osteitis. The effect of gentamicin is considered concentration-dependent, meaning that the highest possible concentration ensures the optimal effect.

The bacterial flora encountered in FRI varies widely, and antimicrobial resistance is increasing [4]. Antimicrobial resistance implies the ineffectiveness of an antimicrobial agent in treating an infection. Susceptibility of bacteria to a specific antibiotic as tested in a clinical setting is based on maximum concentrations that are considered safe upon systemic use [17]. However, through elution of local antibiotics, a much higher local tissue concentration can be reached, which makes the minimum inhibitory concentrations (MICs) more relevant than their interpretation in local FRI treatment.

We asked: (1) What is the in vitro elution pattern of gentamicin from CERAMENT® G using a full washout model? (2) What is the in vitro antimicrobial activity (zone of inhibition) of CERAMENT® G against bacterial isolates found in fracture-related infection with different susceptibility levels toward gentamicin?

## Materials and Methods

### Elution Kinetics of CERAMENT® G

CERAMENT® G was mixed under sterile conditions following the manufacturer’s instructions. Each sample of 10 mL contained 175 mg of gentamicin and was left to cure for 15 minutes in a sterile, prefabricated mold. This created cubical blocks with a surface area of 35 cm^2^. In vitro elution of gentamicin from CERAMENT® G was performed in two setups: one in a saline solution (NaCl 0.9%) and one in human serum.

Five cured blocks of CERAMENT® G were each placed in a separate sterile container. After placement, 100 mL of saline solution was added to four containers. To the fifth container, 100 mL of fresh AB-negative human serum (provided daily by the hospital’s local blood bank) was added. All containers were kept in an incubator with a fixed temperature of 37°C.

After 1, 2, 4, and 8 hours on Day 1, and subsequently every 24 hours until Day 14, at Day 28, and at Day 56, the blocks were transferred to a new sterile container and 100 mL of fresh saline or serum was added. We extracted 1 mL of liquid after stirring the solution of each container for 1 minute on an automated magnetic stirring plate. Samples were stored in 1-mL aliquots in a freezer at -80°C until the analysis was performed on the last day of sampling.

The gentamicin concentration in each sample was analyzed by a gentamicin immunoassay using an Architect C4000, according to the Clinical and Laboratory Standards Institute guidelines. The lower limit of quantification was 0.50 mg/L, and the upper limit was 10 mg/L. Samples of day 1 to 5 were diluted 100-fold.

### Microorganisms

We used the four most-frequently cultured bacterial species found in FRI: *Staphylococcus aureus*, *Staphylococcus epidermidis*, *Pseudomonas aeruginosa*, and *Enterobacter cloacae*. For each species, four different clinical isolates were obtained, each with a different susceptibility to gentamicin. This susceptibility, expressed as the MIC, varied from susceptible to highly resistant (according to the European Committee on Antimicrobial Susceptibility Testing [EUCAST]) (Table 1). The susceptibility of each isolate was classified into four different groups: fully susceptible (MIC 0.064 to 4 mg/L), minimally resistant (MIC 4 to 16 mg/L), moderately resistant (MIC 8 to 96 mg/L), and highly resistant (24 to 1024 mg/L), depending on each organism. The MICs were determined using a gentamicin gradient diffusion test, ETEST® (Biomérieux) [2], and verified by broth microdilution susceptibility testing according to International Organization for Standardisation standard 20776-1:2019 [12].

**Table 1.** Zones of inhibition of CERAMENT® G for different microorganisms

Bacterium	MIC	Disc 1	Disc 2	Disc 3	Disc 4	Disc 5	Mean ± SD
*S. aureus*	< 0.064	38	40	35	37	34	36.8 ± 2.4
*S. aureus*	4	25	25	25	25	25	25.0 ± 0.0
*S. aureus*	32	19	20	20	20	20	19.8 ± 0.4
*S. aureus*	> 1024	0	0	0	0	0	0
*S. epidermidis*	< 0.064	44	45	45	45	43	44.4 ± 0.9
*S. epidermidis*	4	30	30	30	29	30	29.8 ± 0.4
*S. epidermidis*	8	30	29	29	29	28	29.0 ± 0.7
*S. epidermidis*	24	25	25	25	25	25	24.8 ± 0.4
*P. aeruginosa*	4	30	31	31	30	31	30.6 ± 0.5
*P. aeruginosa*	16	23	24	24	24	23	23.6 ± 0.5
*P. aeruginosa*	64	18	18	18	18	17	17.8 ± 0.4
*P. aeruginosa*	256	11	11	12	11	11	11.2 ± 0.4
*E. cloacae*	0.5	31	30	30	30	29	30.0 ± 0.7
*E. cloacae*	4	28	28	27	27	27	27.4 ± 0.5
*E. cloacae*	64	20	19	20	20	20	19.8 ± 0.4
*E. cloacae*	256	12	12	14	12	13	12.6 ± 0.9

MIC = minimum inhibitory concentration.

### Determining the Antimicrobial Activity

To analyze the difference in the efficacy of CERAMENT® G in the same bacterial species between gentamicin-resistant and gentamicin-susceptible bacterial isolates, differences in zones of inhibition (ZOI) were determined after 24 hours of incubation. The antimicrobial activity of CERAMENT® G against the 16 isolates mentioned above was evaluated using a Kirby-Bauer test. Under sterile conditions, 10 mL of CERAMENT® G was prepared according to the manufacturer’s instructions and put into a sterile, prefabricated mold, creating small discs with a surface area of 25 mm^2^.

**Fig. 1:**
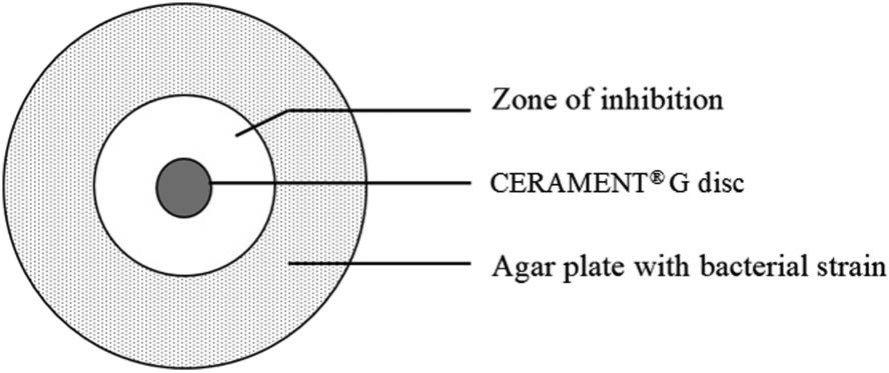
This is a schematic representation of an agar plate populated by a bacterial strain, with the zone of inhibition around a CERAMENT® G disc.

The antimicrobial effect was then determined using the disk diffusion method according to EUCAST [5] using Mueller-Hinton agar plates (Becton, Dickinson and Company), and an inoculum of 1.5 × 108 colony-forming units/mL, that is, 0.5 McFarland. With subcultures of each microorganism, 80 plates were inoculated, with five plates per test setting. Under sterile conditions, a disc of CERAMENT® G was placed in the center of each plate before being incubated at 37°C for 24 hours. After incubation, the ZOI was measured in mm, according to the EUCAST protocol (Fig. 1).

### Outcome Measures

The primary outcome of elution testing was the concentration of gentamicin in mg/L. The primary outcome of antimicrobial activity testing was the ZOI in mm.

### Ethical Approval

Ethical approval was not sought because well-documented in vitro testing was performed to answer the study questions.

### Statistical Analysis

All analyses were conducted using IBM SPSS Statistics for Windows (version 24.0, IBM Corp). ZOIs for groups of isolates with the same level of resistance are reported as a mean and standard deviation. ZOIs are reported for four groups of isolates (that is, susceptible, minimal resistance, moderate resistance, and high resistance). The ZOI was compared within the same bacterial species between each bacterial isolate using a one-way analysis of variance with a post hoc pairwise independent t-test. A p value < 0.05 (two-sided) was considered statistically significant. Nonlinear regression statistics were calculated using Prism (version 9.5, Graphpad Software).

## Results

### Elution Kinetics of CERAMENT® G

**Fig. 2:**
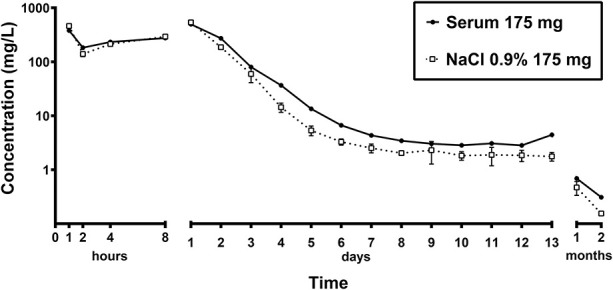
This graph depicts the elution of gentamicin from CERAMENT® G. After the first 24 hours, 95% of all gentamicin was eluted. Gentamicin was still detectable after 2 months.

Gentamicin showed rapid elution owing to CERAMENT® G in human serum and saline. After the first hour, a gentamicin concentration of 381 mg/L was found in human serum and 460 ± 12 mg/L was found in saline. This increased during the first 24 hours to a peak of 497 mg/L in human serum and 537 ± 10 mg/L in saline. After 24 hours, 95% of all gentamicin was eluted in the human serum and saline samples. The concentration dropped below an MIC of 4 mg/L after 8 days in human serum (3.5 mg/L). In saline, the concentration dropped below an MIC of 4 mg/L after 6 days (3.3 ± 0.4 mg/L). After 1 and 2 months, gentamicin was still detectable in human serum and saline, with values below 1 mg/L (Fig. 2).

### Antimicrobial Activity of CERAMENT® G

**Fig. 3:**
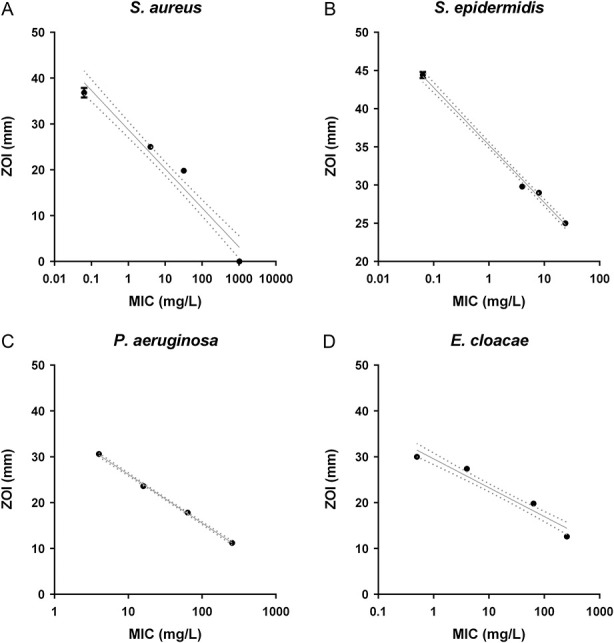
Pictured in these graphs is the effect of CERAMENT® G on bacterial isolates with different MICs toward gentamicin. (**A**) Shows the effect on *Staphylococcus aureus*, (**B**) shows the effect on *Staphylococcus epidermidis*, (**C**) shows the effect on *Pseudomonas aeruginosa*, and (**D**) the effect on *Enterobacter cloacae*. Lines depicted are nonlinear regressions with 95% CI. ZOI = zone of interest; MIC = minimum inhibitory concentration.

Of all 16 isolates, only *S. aureus*, with an MIC > 1024 mg/L, showed no ZOI. For all four species tested, ZOI values varied from 11 to 45 mm and were inversely correlated with the MIC; the higher the MIC, the less the antimicrobial effect of CERAMENT® G. One-way analysis of variance results were significant, with p < 0.0001 for the change in effect and a higher MIC in each of the studied groups. The ZOIs in *S. epidermidis*, with an MIC of 4 and 8, did not differ (p = 0.07) (Fig. 3). The effect of gentamicin was statistically reduced for each increasing MIC. This reduction, that is, the regression coefficient, was -8.5 mm (95% confidence interval -7.5 to -9.5) for *S. aureus*, -7.5 mm (95% CI -7.1 to -7.9) for *S. epidermidis*, -10.6 mm (95% CI -10.2 to -11) for *P. aeruginosa*, and -6.2 mm (95% CI -5.5 to -7.1) for *E. cloacae*.

## Discussion

To delay and prevent new biofilm formation in the treatment of FRI, antibiotics must reach high local levels within the first 24 hours after surgery. These local levels of antibiotics, which are reached through local elution, may also be effective against bacteria that are not susceptible to levels reached through systemic administration. In this study, in vitro elution of gentamicin from CERAMENT® G showed very high release within the first 24 hours and antimicrobial activity against bacterial isolates found in FRI that were systemically untreatable with gentamicin. This finding raises the question of whether EUCAST cutoff points for systemic application are useful for the local use of CERAMENT® G. A standardized method for testing the effectiveness of local antibiotics is needed to predict the clinical outcome of FRI treatment.

### Limitations

First, the method of determining the elution of gentamicin from CERAMENT® G is open for discussion. In this experiment, we used a full washout method, replacing the entire medium after taking concentration samples. In other elution models, only 10 mL (20%) of the total volume was replaced by fresh medium, stacking gentamicin concentrations for the remaining study period. When replacing a larger volume of elution medium, a higher-concentration gradient arises between the CERAMENT® G and its containing medium, therefore promoting a larger concentration of gentamicin to elute. This has been demonstrated in various elution models comparing in vitro versus in vivo antibiotic elution from polymethyl methacrylate spacers [13]. When comparing these models, gentamicin levels showed a rapid decrease after 30 hours in the drainage fluid of patients who were surgically treated for hip infection, reflecting in vitro models, although local tissue samples were not obtained. It thus remains unclear which in vitro elution model best reflects the clinical situation. Second, the gentamicin concentration was analyzed using a gentamicin immunoassay on an Architect C4000. This method has a lower limit of quantification of 0.5 mg/L. Concentrations of gentamicin analyzed 2 months after the start of the elution experiment were 0.31 mg/L in serum and 0.16 ± 0.01 mg/L in saline. These values are below the lower limit of quantification and therefore are not reliable. Finally, the assessment of antimicrobial activity in this study was based upon acute effectiveness in a cultured microorganism. This might not represent the activity of CERAMENT® G in vivo, because it is clinically used to prevent or treat FRI. It is long known that biofilms can still form on antibiotic-loaded polymethyl methacrylate implants, decreasing antibiotic efficacy, but the clinical relevance of these findings is unknown. A reliable in vitro or in vivo model of antibiotic effectiveness on biofilms is unavailable; thus, it is difficult to draw clinically useful conclusions [3, 24, 26].

### Elution Kinetics of CERAMENT® G

In this study, nearly all of the gentamicin was eluted within the first 24 hours, but levels were still detectable after 2 months. In earlier models, it took 2 to 4 days to elute this amount [14, 16, 25]. In the current study, in vitro elution of gentamicin from CERAMENT® G showed a similar elution curve as in earlier published work [14]. Overall, the initial gentamicin release is 100 to 1000 times above the MIC in each setting, high enough to prevent biofilm formation and eradicate susceptible bacteria [10].

Gentamicin was still measurable after a longer period of time in both mediums. Although gentamicin was still measurable after 2 months, its antimicrobial effect should not be diminished because the antibiotic stability of aminoglycosides is excellent over longer periods of time [23]. Prolonged gentamicin concentrations below the MIC could induce antibiotic resistance over time. However, scientific evidence regarding increased antimicrobial resistance as a result of local antibiotic use is lacking [26]. Systemic toxicity when using local antibiotics has not been shown in any clinical study, but scientific evidence is scarce [15, 27]. The use of CERAMENT® G in vivo showed no systemic toxicity [25]. Additionally, the histopathologic effects of CERAMENT® G have been studied, with no local toxic effect but potential osteoconductive properties in the long term [7, 22].

### Antimicrobial Activity of CERAMENT® G

CERAMENT® G showed antimicrobial activity against *S. aureus* (except *S. aureus* with an MIC > 1024 mg/L), *S. epidermidis, P. aeruginosa,* and *E. cloacae*. There was no difference in ZOI between fivefold repetitions of testing bacterial isolates with the same MIC within the same species, indicating an accurate test. The current EUCAST clinical cutoff MICs for gentamicin are 1 mg/L for *S. aureus* and for *S. epidermidis* and 4 mg/L for *P. aeruginosa* and *E. cloacae* [6]. These cutoffs are based on the likelihood of therapeutic success by systemic application with a standard dosing regimen. Considering this, local application of gentamicin via CERAMENT® G can still be effective against organisms with an MIC > 4 mg/L. In this experiment *S. aureus*, *S. epidermidis, P. aeruginosa*, and *E. cloacae* with an MIC above the EUCAST clinical cutoff still showed a ZOI, indicating that local gentamicin administration could be useful to treat pathogens otherwise resistant to systemically reachable levels. This means that local antibiotic administration in case of systemically resistant bacteria could still be useful in FRI treatment.

### Conclusion

CERAMENT® G is capable of releasing high levels of gentamicin in a short period of time, high enough to prevent biofilm formation and eradicate susceptible bacteria. This study shows that CERAMENT® G has antimicrobial activity against bacterial isolates that are resistant to gentamicin when systemically administered, and confirms that the cutoff points for systemic application of gentamicin are not very useful for the local use of CERAMENT® G. The in vitro effect is clear, but reliable in vivo biofilm models are needed to evaluate the effect of local antibiotics in clinical settings. Additionally. in vivo efficacy and safety studies are required to assess the cytotoxicity of these high local antibiotic levels. Finally, a standardized method, such as the EUCAST disk diffusion method, should be developed, representing the in vivo situation of FRI. This will ultimately lead to reliable FRI treatment prediction models for the use of local antibiotics.

## References

[1] Bezstarosti H Metsemakers WJ van Lieshout EMM Management of critical-sized bone defects in the treatment of fracture-related infection: a systematic review and pooled analysis. Arch Orthop Trauma Surg. 2021;141:1215-1230. 32860565 10.1007/s00402-020-03525-0 PMC8215045

[2] Biomerieux USA. Etest improving therapeutic decisions. Available at: https://www.biomerieux-usa.com/clinical/etest. Accessed November 7, 2023.

[3] Bjarnsholt T Alhede M Alhede M The in vivo biofilm. Trends Microbiol. 2013;21:466-474. 23827084 10.1016/j.tim.2013.06.002

[4] Dickey SW Cheung GYC Otto M. Different drugs for bad bugs: antivirulence strategies in the age of antibiotic resistance. Nat Rev Drug Discov. 2017;16:457-471. 28337021 10.1038/nrd.2017.23 PMC11849574

[5] European Committee on Antimicrobial Susceptibility Testing. Antimicrobial susceptibility testing EUCAST disk diffusion method, version 7.0. Available at: http://www.eucast.org/fileadmin/src/media/PDFs/EUCAST_files/Disk_test_documents/2019_manuals/Manual_v_7.0_EUCAST_Disk_Test_2019.pdf. Accessed August 28, 2023.

[6] European Committee on Antimicrobial Susceptibility Testing. Breakpoints for interpretation of mics and zone diameters. Available at: http://www.eucast.org/fileadmin/src/media/PDFs/EUCAST_files/Breakpoint_tables/v_9.0_Breakpoint_Tables.pdf. Accessed November 7, 2023.

[7] Ferguson J Athanasou N Diefenbeck M McNally M. Radiographic and histological analysis of a synthetic bone graft substitute eluting gentamicin in the treatment of chronic osteomyelitis. J Bone Jt Infect. 2019;4:76-84. 31011512 10.7150/jbji.31592 PMC6470655

[8] Hake ME Young H Hak DJ Stahel PF Hammerberg EM Mauffrey C. Local antibiotic therapy strategies in orthopaedic trauma: practical tips and tricks and review of the literature. Injury. 2015;46:1447-1456. 26007616 10.1016/j.injury.2015.05.008

[9] Hellebrekers P Verhofstad MHJ Leenen LPH Varol H van Lieshout EMM Hietbrink F. The effect of early broad-spectrum versus delayed narrow-spectrum antibiotic therapy on the primary cure rate of acute infection after osteosynthesis. Eur J Trauma Emerg Surg. 2020;46:1341-1350. 31312856 10.1007/s00068-019-01182-6 PMC7691296

[10] Howlin RP Brayford MJ Webb JS Cooper JJ Aiken SS Stoodley P. Antibiotic-loaded synthetic calcium sulfate beads for prevention of bacterial colonization and biofilm formation in periprosthetic infections. Antimicrob Agents Chemother. 2015;59:111-120. 25313221 10.1128/AAC.03676-14 PMC4291338

[11] Iliaens J Onsea J Hoekstra H Nijs S Peetermans WE Metsemakers WJ. Fracture-related infection in long bone fractures: a comprehensive analysis of the economic impact and influence on quality of life. Injury. 2021;52:3344-3349. 34474918 10.1016/j.injury.2021.08.023

[12] International Organization for Standardization. Susceptibility testing of infectious agents and evaluation of performance of antimicrobial susceptibility test devices -- part 1: broth micro-dilution reference method for testing the in vitro activity of antimicrobial agents against rapidly growing aerobic bacteria involved in infectious diseases. Available at: https://www.iso.org/standard/70464.html. Accessed November 7, 2023.

[13] Kelm J Regitz T Schmitt E Jung W Anagnostakos K. In vivo and in vitro studies of antibiotic release from and bacterial growth inhibition by antibiotic-impregnated polymethylmethacrylate hip spacers. Antimicrob Agents Chemother. 2006;50:332-335. 16377705 10.1128/AAC.50.1.332-335.2006 PMC1346773

[14] Lindberg F. Antibiotic elution and bone remodelling with a novel bone substitute impregnated with gentamicin. In: European Bone and Joint Infection Society (EBJIS). Montreux, Switzerland; 2012.

[15] Luu A Syed F Raman G Two-stage arthroplasty for prosthetic joint infection: a systematic review of acute kidney injury, systemic toxicity and infection control. J Arthroplasty. 2013;28:1490-1498.e1491. 23578491 10.1016/j.arth.2013.02.035

[16] Maier GS Roth KE Andereya S In vitro elution characteristics of gentamicin and vancomycin from synthetic bone graft substitutes. Open Orthop J. 2013;7:624-629. 24285988 10.2174/1874325001307010624 PMC3841967

[17] Matuschek E Brown DF Kahlmeter G. Development of the EUCAST disk diffusion antimicrobial susceptibility testing method and its implementation in routine microbiology laboratories. Clin Microbiol Infect. 2014;20:O255-266. 24131428 10.1111/1469-0691.12373

[18] McNally M Nagarajah K. Osteomyelitis. Mini syposium: pathology. Orthop Trauma. 2010;24:416-429.

[19] McNally MA Ferguson JY Lau AC Single-stage treatment of chronic osteomyelitis with a new absorbable, gentamicin-loaded, calcium sulphate/hydroxyapatite biocomposite: a prospective series of 100 cases. Bone Joint J. 2016;98:1289-1296. 27587534 10.1302/0301-620X.98B9.38057

[20] Metsemakers WJ Morgenstern M Senneville E General treatment principles for fracture-related infection: recommendations from an international expert group. Arch Orthop Trauma Surg. 2020;140:1013-1027. 31659475 10.1007/s00402-019-03287-4 PMC7351827

[21] Morgenstern M Vallejo A McNally MA The effect of local antibiotic prophylaxis when treating open limb fractures: a systematic review and meta-analysis. Bone Joint Res. 2018;7:447-456. 30123494 10.1302/2046-3758.77.BJR-2018-0043.R1 PMC6076360

[22] Oliver RA Lovric V Christou C Walsh WR. Comparative osteoconductivity of bone void fillers with antibiotics in a critical size bone defect model. J Mater Sci Mater Med. 2020;31:80. 32840717 10.1007/s10856-020-06418-1 PMC7447650

[23] Samara E Moriarty TF Decosterd LA Richards RG Gautier E Wahl P. Antibiotic stability over six weeks in aqueous solution at body temperature with and without heat treatment that mimics the curing of bone cement. Bone Joint Res. 2017;6:296-306. 28515059 10.1302/2046-3758.65.BJR-2017-0276.R1 PMC5457644

[24] Sonderholm M Bjarnsholt T Alhede M The consequences of being in an infectious biofilm: microenvironmental conditions governing antibiotic tolerance. Int J Mol Sci. 2017;18:2688. 29231866 10.3390/ijms18122688 PMC5751290

[25] Stravinskas M Horstmann P Ferguson J Pharmacokinetics of gentamicin eluted from a regenerating bone graft substitute: in vitro and clinical release studies. Bone Joint Res. 2016;5:427-435. 27678329 10.1302/2046-3758.59.BJR-2016-0108.R1 PMC5047051

[26] van Vugt TAG Arts JJ Geurts JAP. Antibiotic-loaded polymethylmethacrylate beads and spacers in treatment of orthopedic infections and the role of biofilm formation. Front Microbiol. 2019;10:1626. 31402901 10.3389/fmicb.2019.01626 PMC6671866

[27] Walenkamp GH Vree TB van Rens TJ. Gentamicin-pmma beads. Pharmacokinetic and nephrotoxicological study. Clin Orthop Relat Res. 1986;205:171-183. 3516500

